# Without a Debate on Sufficiency, a Circular Plastics Economy will Remain an Illusion

**DOI:** 10.1007/s43615-022-00240-3

**Published:** 2022-12-14

**Authors:** Linda Mederake

**Affiliations:** Ecologic Institute, Berlin, Germany

**Keywords:** Circular economy, Plastics, Plastic prevention, Recycling, Sufficiency, Plastics regulation

## Abstract

To tackle the “plastic crisis”, the concept of circular economy has attracted considerable attention over the past years, both by practitioners and scholars alike. Against this background, this article reflects from a political scientist’s point of view on key issues currently under discussion regarding the implementation of a circular plastics economy. To do so, the article covers issues raised at the 2021 edition of the *Tutzing Symposion*, an annual event organized by DECHEMA, the German Society for Chemical Engineering and Biotechnology. These issues include renewable feedstock, life cycle assessments, chemical recycling, appropriate regulatory frameworks, and the allocation of responsibilities to curb plastic pollution. In addition, the article draws on mainly social scientists’ research to point out limitations of the most common understanding of the circular economy concept which describes the circular economy as a holistic approach that enables eco-economic decoupling and thus prevents ecological collapse while still allowing for economic growth. As a result, the article calls for a paradigm shift that challenges this popular, technocratic vision of the circular economy and puts forward sufficiency, i.e., an absolute reduction in production and consumption, as a prerequisite for the realization of a truly circular plastics economy.

## Introduction

Plastics have become an integral part of our lives. Whether as high-performance materials in the aviation and automotive industries, as functional and construction materials for electronic devices, or in everyday items such as packaging and clothing, plastics are everywhere. What is more, plastics are an essential and often clearly superior material in many applications, as, for instance, for medical supplies. At the same time, the term “plastic crisis” is gaining popularity, with public attention drawn in particular to marine plastic pollution and its environmental consequences as well as the ubiquity of microplastics in our environment.

Against the background of this ambiguity, the 59th *Tutzing Symposion* was held in October 2021 under the title “Polymers for a better life and circular economy” [1]. The *Tutzing Symposion* is an annual event organized by DECHEMA, the German Society for Chemical Engineering and Biotechnology. Its 2021 edition^1^ covered key issues currently under discussion — among scientists and industry, but also in the wider public — with regard to a circular plastics economy, including renewable feedstock, life cycle assessments (LCAs), chemical recycling, appropriate regulatory frameworks, and the allocation of responsibilities to curb plastic pollution. In addition, the symposium touched on the scholarly debate regarding the limits of the circular economy concept. While these limits are not widely acknowledged to date, they are discussed by some (social) scientists.

As the DECHEMA organized the symposium, most speakers and participants were either polymer scientists or representatives of the polymer industry. However, the organizers of the symposium aimed to foster exchange and a broader discussion on the public perception, sustainability, as well as the advantages and disadvantages of plastics. Therefore, they specifically invited several speakers from other academic disciplines, civil society, and the political arena.

This article reflects from a political scientist’s point of view^2^ on the topics discussed at the symposium and argues for sufficiency, i.e. “a transition to a society where we manage with (in some cases much) less” [5], as a prerequisite for the realization of a truly circular plastics economy. This includes “reconsidering superfluous consumption, reconnecting consumption to well-being and satisfaction of needs and improving self-sufficiency at the system level (e.g., refusing to produce or buy, producing and buying local)” [6]. As a society-wide approach, sufficiency focuses on how to reduce production and consumption in absolute terms relying not primarily on technology, product, and process innovations, but stricter policies, new business models, as well as civil society actions and education to reshape industry and society with the aim to tackle the root causes of unsustainability [5]. Although the sufficiency approach has gained some support in the scientific literature more recently [e.g., 7–12], these articles are still exceptions. The political debate, as well, is still dominated by a circular economy narrative that focuses on technological innovation and eco-efficiency [13].

This article sets out to explain why a paradigm shift is needed that challenges the technocratic vision of the circular economy and promotes an understanding of circularity based on the principle of sufficiency. To do so, the article outlines major challenges of the plastics sector in the following section, before it turns to the contributions that recycling (“Industry Focuses on New Recycling Technologies to Tackle the Plastic Problem” and “We Cannot Recycle Our Way Out of the Crisis — Plastic Prevention Is Needed” sections) and individual actions (“The Attainable Impact of Individual Plastic Prevention Is Very Limited” section) provide to tackle the plastics problem. These sections reveal that strengthening secondary raw materials markets and individual action against plastic pollution will not suffice considering the major challenges faced by the plastics industry and society as a whole.

The circular economy concept is often portrayed as a holistic approach able to tackle the plastic crisis successfully, while still allowing for economic growth. Drawing on (mainly) social scientists’ research, this article challenges this assumption and points out limitations of this popular understanding of the circular economy concept (“There Are Material and Political Limits to a Circular (Plastics) Economy” section). The findings substantiate calls for a reduction in production and consumption of plastics, i.e. a sufficiency approach. Last, but not least, the article takes a closer look at existing EU policies that promote a circular plastics economy only to find that current policies fall short of expectations with regard to plastic prevention (“Existing Policies Do Not Put Sufficient Emphasis on the Need to Reduce Plastic Production” section). The article thus concludes with a call for sufficiency as a prerequisite for the realization of a circular plastics economy (“The Way Ahead: Sufficiency as a Prerequisite for a Circular Plastics Economy” section).

## The Plastics Sector Faces Enormous Challenges

The plastics sector is facing multiple challenges, of which three received particular attention at the *Tutzing Symposion*: Plastics in the environment, the climate crisis, and finite resources. For plastics in the environment, there is fundamental agreement among stakeholders along the plastics life cycle that plastics do not belong in the environment — neither as macroplastics nor as microplastics — and that appropriate efforts have to be taken to prevent their entry into the environment. Nevertheless, plastic leakage is still increasing and has recently been judged to have exceeded the save operating space for humanity as part of the planetary boundary for novel entities^3^ [14]. Climate change is also broadly recognized as a challenge, with industry representatives frequently emphasizing the contribution of plastics in the fight against global warming. In contrast, scientific publications from social scientists, but also civil society actors emphasize the plastics industry’s dependence on fossil raw materials, which is increasingly becoming a problem in light of ambitious climate goals such as greenhouse gas neutrality [cf., for instance, 11, 15–17]. Nevertheless, the public debate often focuses on littering issues — drawing attention away from the unsustainability of using fossil feedstock [18].

Against the background of climate change, the *Tutzing Symposion* evidenced that current discussions are concerned with the calculation and comparison of life cycle assessments (LCAs) for plastic packaging and plastic-free alternatives. Here, plastic packaging frequently performs very well compared to alternatives [cf., for instance, a study of grocery shopping bags by 19]. However, every LCA is based on a number of assumptions including the choice of factors to consider, the weighting of factors as well as the assigned values. These assumptions significantly alter results. Moreover, most LCAs comparing plastics with alternative materials published today only consider the cradle-to-factory gate phases of the production. Narrowing down the scope and excluding the end-of-life phase results in analyses that are grossly misleading [20, 21]. It is therefore crucial to cover the post-use or end-of-life stages of a product in LCAs. In general, the assessment should encompass the social, economic, and environmental impacts of waste production and management. This includes the effects of littering, of plastics and microplastics entering the environment [22], and the role of (improved) solid waste management [20]. As there is no recognized or agreed method yet to include the leakage of plastics into the environment, the subject should at least be discussed within respective LCA studies [21]. In the medium term, binding standards for LCAs could improve comparability of assessments. Indeed, experts present at the *Tutzing Symposion* anticipated greater standardization in this area in the coming years.

In view of the problems associated with the use of fossil raw materials, the industry is increasingly focusing on alternative, renewable feedstocks [23]. These include, for instance, sugar cane and corn. However, their use for plastics production is often viewed critically due to the pressure on arable land, competing use interests of food crops, as well as the intensive use of fertilizers, pesticides, and water [24]. Some companies are therefore turning to waste products in their search for alternative raw materials. One example is LyondellBasell, which is testing the use of vegetable oil waste (used cooking oil) and residual oils [25]. Nevertheless, it is unrealistic to expect that the entire plastic production could be switched to renewable raw materials as feedstock [26]. That is why politicians and business representatives highlight the importance to keep plastics in the cycle and strengthen the secondary raw materials market.

## Industry Focuses on New Recycling Technologies to Tackle the Plastic Problem

One way to keep plastics in the cycle and strengthen the secondary raw materials market is to increase recycling rates. To do so, some of the largest polymer producers worldwide have rather recently been moving into investing and the scaling up of new recycling technologies [27]. As a result, research funds from major chemical companies are now flowing into the recycling sector, which has traditionally been dominated by small and medium-sized enterprises.

As a complement to the traditional mechanical recycling, companies as well as applied research institutes present at the *Tutzing Symposion* strongly argued in favor of chemical (or feedstock) recycling. This term describes any reprocessing technology that uses chemical agents or processes to break down plastics into monomers, oligomers or higher hydrocarbons that can then be used to make new plastics or other materials (e. g., fuels) [28].^4^ The advantages of chemical recycling put forward by industry associations [32] and mentioned in the existing literature [see, for instance, 33–35] include the recovery of material quality, alternative raw material production, and support for the closed-loop circulation of plastics. Chemical recycling is further portrayed to have a huge potential to reduce the existing recycling gap for materials that cannot be recycled mechanically today [33]. These include, for instance, plastics such as films and multimaterials, and plastics that have been contaminated [35, 36]. It is argued that in doing so, chemical recycling could live up to the current ambition to close the loop on plastics [35].

Yet, chemical recycling is a controversial technology and its environmental benefits are intensely debated [33, 34] — a fact that has not been acknowledged at the *Tutzing Symposion*. Especially environmental NGOs, such as Zero Waste Europe, European Environmental Bureau (EEB), and ECOS, believe the industry’s claims to be exaggerated [33, 37, 38]. They contest the proposition that chemical recycling can cope with mixed, contaminated plastic waste [36] and highlight methodological weaknesses in LCA studies developed by, or in affiliation with, businesses [39]. Critics raise several concerns about chemical recycling:

1. They fear that investments could generate “lock-in effects” and “path dependency”. This is to say, municipalities that buy into the model usually have to stick with it for many years due to large amounts of money invested and contractual agreements to provide certain quantities of waste over a defined period of time. This can lead to perverse incentives not to decrease plastic waste generation. An example for such a case was observed in Oregon, where the presence of a pyrolysis plant was used to argue against a partial ban on polystyrene [28].
2. Chemical recycling has high energy requirements, leading to greenhouse gas (GHG) emissions that are, according to estimates, 110% higher than those of mechanical recycling [28].^5^ At present, chemically recycled plastics can even have a higher level of embedded carbon than virgin plastics. Moreover, the level of GHG emissions depends on the decarbonization of electricity. If the electricity sector fails to meet expected decarbonization targets, emissions from chemical recycling would be even higher than predicted. The development and scale-up of chemical recycling should thus be contingent on the decarbonization of energy sources [28]. Moreover, it is important to keep in mind that electricity generation from renewable energies is resource-consuming as well (through the necessary construction of solar panels, wind turbines, etc.), so that even renewable energies are not infinitely available.
3. Chemical recycling processes, particularly for certain types of plastics such as polyester and PVC, generate harmful waste streams [37]. Yet, industry experts often downplay these (and similar) issues of toxicity and portray them as purely technological hurdles. This has environmental justice implications, as the most polluting petrochemical factories around the world are located near low-income communities with high percentages of their residents from ethnic minorities and the working class. Residents and workers in many of these “petrochemical communities” struggle with pollution and environmental hazards [11].
4. Chemical recycling could risk diverting corporate and public attention as well as research and development financing away from better, more efficient solutions. These include more sustainable reuse solutions as well as plastic product design that facilitates mechanical recycling [28].

All things considered, chemical recycling is still in the early stages of development, struggles to be economically viable [40] and, thus, has not yet been proven at scale. Hence, there is not enough data to support solid economic feasibility analyses of different chemical recycling technologies [35] and it is still too early to make accurate assumptions about its impacts and future contributions to solve the plastic problem [28].

## We Cannot Recycle Our Way Out of the Crisis — Plastic Prevention is Needed

At the *Tutzing Symposion* it became apparent that it is still common, especially among industry representatives, to reduce the plastic problem to that of proper waste management, or at least to focus on this issue when it comes to discussing solutions [cf. also 41]. Yet, we will not be able to solve the plastic problem by simply improving the collection and recycling infrastructure. This is because of the increasing and even accelerating virgin plastic production which is expected to increase by 40 per cent over the next decade [28]. Increased waste management capacity alone will just not be able to keep pace with projected growth in plastic production and waste generation [28, 42].

The general problem with a focus on downstream measures is that they reinforce the status quo: Major petrochemical companies worldwide drive the capacity growth in plastic production with large-scale investments in new refineries, steam crackers, and production plants. With these investments, plastic production is essentially becoming the new engine of growth for the petrochemical industry. This raises strong concerns with critics, but also worries about the creation of a “plastic bubble” whereby new investments risk to become stranded assets [28].

It is thus necessary to focus on upstream measures that prevent plastic waste and reduce absolute plastic consumption per person and by society. A stronger focus on upstream measures necessitates a change towards business models that focus on slowing and closing resource loops, for instance, through repair and reuse, product service systems, and new delivery models [5, 28, 43]. Such business models do not rely on the idea to sell more and more products. For long-lived products, for example, leasing, rental, or performance contracting enable companies to operate economically without creating incentives to always buy new, short-lived products. These business models also “offer new ways to engage customers to establish and strengthen long-term relationships” [44]. The fear of cannibalizing old, proven business models was cited at the symposium as the main obstacle for changing business models. In addition, for larger companies, new business models often serve too much of a niche to have the potential to directly replace an old business model. Therefore, new business models are usually tested in start-ups, subsidiaries, or spin-offs.

## The Attainable Impact of Individual Plastic Prevention is Very Limited

In the public debate about the plastics problem, many people still refer to the individual responsibility of citizens to reduce their plastic consumption, to buy the “right”, meaning environmentally friendly, products and to dispose of waste properly, i.e. as prescribed by the local waste management system [cf., for instance, 45]. Similarly, some presentations at the *Tutzing Symposion* emphasized the role of consumer education to tackle the plastics problem. However, research shows that consumers are simply overtaxed with the “right” purchasing decisions [46, 47], as well as many disposal decisions [48]. What is more, it is quite common that consumers receive contradictory information about the sustainability of products [see, for instance, 49]. At the same time, it is almost impossible for them to check the sustainability claims of manufacturers and to gain an overview of the validity of various labels. In the scientific debate about the discrepancy between attitudes and actions of the general public, it has therefore been described many times that it is simply wrong to assume that more knowledge is the key to more sustainable consumption [12]. Instead, consumers need clear (economic) incentives, as for instance in pay-as-you-throw systems [50].

Apart from the information and decision-making overload, the “right” behavior of citizens also fails due to many, often structural and system-inherent barriers [51, 52]. For example, it requires additional effort to adopt new habits for the purchasing, use, and disposal of plastic products. Whether individuals can make this extra effort depends, among other things, on their time and financial resources, human and social capital, skills, and abilities, which are not evenly distributed across the population [12, 53]. In addition, the amount of plastic (packaging) used in the production, distribution, and transport of many products is significantly higher than the plastic packaging visible in stores. In these cases, consumers can only influence a small portion of the plastic waste volume, e.g., the plastic carrier bag. Hence, plastic prevention on an individual level can only reduce plastic waste to a small extent, while far greater scope for action and responsibility lies with industry and politics [48]. This exemplifies the need for system-level changes that involve actors across government, industry, and civil society to achieve the necessary absolute reductions of plastic waste volumes.

When it comes to responsibility, it is also important to note that we make things too easy for ourselves if we reduce the plastic problem mainly to plastic waste leaked into the environment in the Global South. This is because a portion of plastic waste from Europe is exported to the Global South, where it ends up polluting the environment [54], but also exacerbates human health problems related to unsafe recovery systems, thus causing key social and environmental justice concerns [55]. Moreover, “there should be humility and greater responsibility taken for past and present mistakes” on the part of the Global North, including for the production and the manufacturing of demand for unsustainable and avoidable plastic products (Alice Mah, Warwick University at the *Tutzing Symposion*).

The circular economy concept is often portrayed as a holistic approach to combine downstream with upstream measures and tackle the plastic crisis successfully. Therefore, we now turn to the concept to see whether these promises hold in practice.

## There Are Material and Political Limits to a Circular (Plastics) Economy

Circular economy has become the “go-to concept” in nearly all discussions to tackle the plastic problem. It has caught attention by all relevant stakeholders in recent years, including academia, businesses, NGOs, and governments [4]. Until today, the definition, objectives, and forms of implementation of the circular economy are still under debate. Nevertheless, the circular economy is usually portrayed to enable eco-economic decoupling to prevent ecological collapse by means of economic and technological innovations as well as new business models that close resource loops while still allowing for economic growth [4, 6, 56–58]. This narrative is common in government policies, including the EU, in corporate strategies and among business consultancies, as well as some international organizations such as the World Economic Forum, the International Resource Panel, and the OECD [4, 59].

Along the same narrative, the participating speakers from industry and (applied) research at the *Tutzing Symposion* expressed great confidence in technical progress and great optimism about being able to jointly solve the identified challenges of the plastics sector through a consequent implementation of a circular economy. Yet, this popular framing of the circular economy concept fails to recognize several challenges and limitations, such as entropy, biophysical limits, rebound effects, social implications, and technological limits [4, 6, 60].

Being subject to the principles of the second law of thermodynamics — that is the inevitability of entropy — recycling requires energy which again generates waste and emissions. Moreover, materials degrade in quantity and quality each time they are cycled or used [61]. A perfect circular economy, where all inputs come from recovered or renewable materials would thus require a general reduction in material demand and economic throughput [4, 60].

Individual circular economy projects and measures should be carefully assessed for their net (global) contribution to sustainability to ensure that circularity interventions actually reduce the pressure on the Earth’s biophysical limits and do not have negative social implications. However, this is not an easy task. In fact, efficiency gains often lead to higher levels of overall resource consumption in the economy (the so-called rebound effect) [9, 62]. Moreover, problems are often shifted along the product life cycle or value chain, i.e., environmental and social improvements in one place lead to problems elsewhere. Vulnerable and marginalized populations in the Global South suffer from these displacements in particular [63]. Unfortunately, there is a general lack of discussion regarding social and environmental justice aspects in most circular economy definitions [58].

The biophysical limits and challenges in terms of social and environmental justice are further exacerbated by technological limits, i.e., the path dependency and lock-in effects of technologies and infrastructures [60]. At present, hundreds of billions of dollars are being invested in virgin plastic production plants, increasing lock-in effects into a business-as-usual trajectory. These investments make a system change ever more urgent [28].

Hobson and Lynch [9] argue that the common conceptualization of the circular economy should be subject to more radical critique and scrutiny if the concept is to bring about the profound changes that its proponents claim are within our collective reach [cf. also 64]. Thus, while the common understanding of the circular economy may seem radical to many business advocates — and is often portrayed as such by advocates — implementation measures taken today are characterized by incremental changes instead of a paradigm shift [65]. That is, because the implementation of a circular economy is usually based on a weak understanding of sustainability and thus fails to address the roots of the problems it claims to address [12, 53]. This is what Mah (2021) calls the paradox of the circular economy: for all its flaws, it has the potential to transform our global economy in ways that could minimize or even eliminate the negative impacts of current economic practices. To do so, the public debate would need to be refocused to include a more radical understanding of the circular economy, combining efficiency improvements with sufficiency approaches [cf. also 66 for a similar argument]: It would mean reducing the production and consumption of single-use plastics and making eco-design of plastic products mandatory which ensures that the products can be reused (or refilled) cost-effectively, safely, and efficiently. It would also mean to acknowledge the dilemmas and unintended consequences of potential “solutions”, particularly in terms of environmental justice. However, Mah sees a high risk that the concept of the circular economy will continue to be diluted if companies succeed in setting the economic and technological course [11].

## Existing Policies Do Not Put Sufficient Emphasis on the Need to Reduce Plastic Production

Many important approaches exist on the policy and regulatory side to prevent plastic waste from entering the environment and to reduce the amounts of plastic waste by increasing circularity [see 67–69]. With regard to policies that explicitly aim for a circular plastics economy, the EU strives for global policy leadership on the issue [13]. Hence, EU narratives and policies will probably define the character of a substantial portion of global plastics governance in the coming years [13]. Therefore, I focus on EU policies in this section to point out that current policies that promote a circular plastics economy are still characterized by incrementalism and the refashioning of existing policies [13, cf. also 65], but fail to initiate the paradigm shift and transition towards a society that “make[s] do with less” [5] which has been identified as crucial in the previous sections.

Circular economy became a guiding principle for EU environmental and economic policies after the publication of the first Circular Economy Action Plan in 2015 [70] and is now a key component of the European Green Deal [71] and the Coronavirus Recovery Plan of the von der Leyen Commission [72]. In the EU, plastics are regulated in waste policies, chemical policies, marine and environmental policies. In terms of a circular plastics economy, the publication of the EU Plastics Strategy in January 2018 has been a widely acknowledged step [73]. In June 2019, EU Directive 2019/904 on the Reduction of the Impact of Certain Plastic Products on the Environment [74] — better known as the Single-Use Plastics Directive — was adopted as a result of the Plastics Strategy. Among other measures, the Directive bans several single-use plastic items including cotton buds, cutlery, stirrers, plates, and straws. For food containers and beverage cups, member states must take measures to achieve a measurable reduction in consumption by 2026 compared to 2022. Further, the Directive includes separate collection targets for single-use plastic bottles and PET bottles have additional targets regarding their recycled plastic content [74].

In addition to the Single-Use Plastics Directive, back in May 2018, the so-called waste package saw a revision of the Waste Framework Directive (2008/98/EC) and other EU waste legislation to strengthen the transition towards a circular economy. Among the changes adopted are increased targets and additional targets for separate collection and recovery of certain types of waste, including the preparation for reuse and recycling of municipal waste [75]. With further amendments, the Packaging and Packaging Waste Directive (94/62/EC) also increased the previous targets for the recycling of (plastic) packaging waste [76].

In addition, the calculation methods for recycling rates have been harmonized across the EU. Furthermore, EU member states are to introduce economic instruments and other measures to increase the reuse of packaging and reduce the consumption of non-recyclable and redundant packaging. For example, deposit systems could be introduced, or incentives provided for the use of recycled content in packaging.

Despite the framing of the abovementioned policies as circular economy policies, Calisto Friant, Vermeulen, Salomone [70] point out that these policies focus on resource efficiency and technological change, but do little to seriously disrupt linear business-models and practices [cf. also 77]. In fact, most measures and almost all targets only aim to improve recycling rates. The Single-Use Plastics Directive includes some more ambitious policies with product bans as well as consumption reduction measures. Yet, these measures only apply to a limited number of plastic items.

Overall, the EU gives disproportionate attention to technical and economic considerations of circularity, especially compared to cultural aspects and aspects of lifestyle transformation including open-source technologies, promotion of social and solidarity economies, as well as voluntary simplicity lifestyles, challenging overconsumption and materialism, etc. Inequality reductions are only envisaged by better distributing future economic benefits instead of redistributing present wealth [70]. This perspective lacks the essential focus on absolute reduction in plastic production and consumption and references to the necessary structural changes to achieve this [cf. 42].

## The Way Ahead: Sufficiency as a Prerequisite for a Circular Plastics Economy

The root of the “plastic crisis” does not lie in the material itself, but in the way we got accustomed to use plastics. Hence, the transition towards a circular plastics economy is of utmost importance. This transition requires a paradigm shift that challenges the technocratic vision of the circular economy and promotes an understanding of circularity based on the principle of sufficiency. This would entail “a focus on health and well-being rather than monetary outcomes; equality and fairness across society and environment; an intergenerational perspective rather than short-termism; and perhaps most importantly, a collective sense of commitment and responsibility” [5].

The challenge in this transition is to (1) accommodate the high diversity and complexity of the plastics system and to (2) provide a holistic, inclusive, and systemic response to the socio-ecological challenges at hand.

To manage the high diversity and complexity of the plastics system is difficult because plastics are not regulated as a separate policy field, but rather as a complex of issues that cut across a number of policy areas, including waste management, chemical policies, marine and environmental policies, as mentioned above. Accordingly, plastics governance is also influenced by developments and perspectives in these — rather different — policy fields. Against this background, a holistic approach is needed that takes into account trade-offs and synergies between the different policy fields related to plastics. A high degree of strategic coordination is required to ensure that measures from a wide range of policies, including regulations (e.g., eco-design measures), market-based instruments (e.g., taxes on fossil feedstock), investments (to develop waste management infrastructure), as well as innovation and research, are meaningfully intertwined [16].

With regard to the holistic, inclusive, and systemic response, the article has shown that it seems unrealistic to assume that a truly circular (plastics) economy could operate in the context of continued economic growth, due to the inevitability of entropy and the lack of evidence that absolute decoupling can be achieved [4]. A clear focus on the “shorter loops” in the value retention hierarchy, i.e., the strategies of refuse, reduce, reuse/resell, repair, refurbish, remanufacture, and re-purpose, is needed for any realization of a circular plastics economy [4]. Moreover, the transition would require a downscaling of plastic production to stay within the earth’s system boundaries. Here, scientists suggest a cap in virgin plastics production that progressively decreases global production allowance for virgin plastics [78] as part of the global plastics agreement that is to be negotiated until 2024 [79].

A sustainable circular plastics economy will involve far-reaching changes in lifestyles and cultures and will necessitate behavioral changes. This will lead to unavoidable costs and benefits. Therefore, social components are essential to equitably distribute these costs and benefits within society (e.g., through progressive taxation and social policies) [55].

Moreover, the transition towards a truly circular plastics economy requires joint efforts by all actor groups — industry, policymakers, public authorities, civil society, and academia. More particularly, integrating sufficiency into societies needs to involve citizens in co-production processes. Without such participatory processes that create ownership and buy-in, the idea of a sustainable circular plastics economy will remain beyond our common reach [66].

## Footnotes

1 The program and list of speakers can be accessed online, cf. [1].

2 This appears particularly worthwhile in view of the fact that the realization of a circular (plastics) economy requires large-scale behavioral, political, and economic change to address the problem of plastic pollution [2]. This change must be informed by sound and objective natural *and* social sciences. However, at present, there is often a disconnect between the natural science research and the complementary social science research that is necessary to understand the social and political dimensions of the plastic pollution problem [3]. Moreover, social science and humanities research on the circular economy is still limited, compared to contributions from environmental sciences or engineering [4].

3 Novel entities are synthetic chemicals, including plastics.

4 The term “chemical recycling” covers a variety of processes, including solvolysis, pyrolysis, and gasification. The following recent reviews provide more details: [29, 30, 31].

5 It should be noted that data for GHG emissions of chemical recycling technologies is still severely limited. Further transparency and monitoring are needed to improve assessments.
